# Precision immunomedicine

**DOI:** 10.1038/emi.2017.22

**Published:** 2017-04-26

**Authors:** Tianlei Ying, Yumei Wen, Dimiter S Dimitrov

**Affiliations:** 1 Key Laboratory of Medical Molecular Virology of Ministries of Education and Health, School of Basic Medical Sciences, Fudan University, Shanghai 200032, China; 2 Protein Interactions Section, Cancer and Inflammation Program, Center for Cancer Research, National Cancer Institute, National Institutes of Health, Frederick, MD 21702, USA

**Dear Editor,**

Individual differences play a major and, in many cases, critical role in the successful prevention and treatment of disease. Throughout history, attempts to individualize therapies have been made with varying degrees of success. For example, blood typing has been used for more than a century to account for individual differences and make blood transfusions safe. However, the application of individualized therapies has been limited. A major cause of the lack of broader use in the past has been the lack of a comprehensive understanding of the many complex processes in the human body that could affect disease progression, outcomes and associated technologies. With the successes of modern biology and the development of new and improved techniques in recent decades, it is becoming possible to precisely tailor therapies to match individual peculiarities in a discipline known as precision medicine.^1^ Advances in the understanding of the basic biology of the human body in health and disease are evolving at an ever-increasing pace, and, as a result, various efforts are being pursued to extend precision medicine's success from its initial focus on cancer to many other diseases, including diabetes, Alzheimer's, obesity and infectious diseases. Importantly, precision medicine will focus on disease as well as on ways to increase an individual's chances of remaining healthy throughout life.

Currently, a promising field of precision medicine is the application of knowledge of modern immunology in the context of the precision medicine ideology for the prevention and treatment of disease. We termed this approach ‘precision immunomedicine' (PIM). This approach takes into account the individual variability of each person but is based on the mobilization of the body's own immune system or uses components of the immune system to fight disease, including immune cell therapy, therapeutic antibodies, vaccines and immune system modulators.

The concept of ‘precision' has already been utilized in cancer immunotherapy, for example, the use of host, immune system and tumor factors as biomarkers to select the appropriate immunotherapy for human cancer. However, PIM is more general and based on individual properties in totality and not only on biomarkers for the selection of immune system-based approaches. It does not necessarily imply that therapies are being developed uniquely for each individual; the focus is on identifying which approaches will be effective for which patients based on immune-related factors. It also holds promise for improving the efficacy, safety and cost-effectiveness of immunotherapy and, in turn, expanding immunotherapy strategies to the treatment of various diseases beyond cancer, such as infectious diseases.

PIM emphasizes a comprehensive understanding of complex and diverse immune mechanisms in response to treatment and prevention modalities (especially immunotherapy modalities), including immune recognition, response, regulation, memory and effector functions. Understanding individual variability in these immune mechanisms would not only facilitate the identification of critical parameters associated with effective immunity but would also help to divide patient groups into specific populations, a process that can be termed ‘immune subtyping,' eventually leading to the more precise diagnosis, prevention and treatment of disease.

PIM may require systems biology approaches. Network-based system strategies are likely to be of value in understanding the full breadth of immunological correlates of clinical outcomes in different individuals. For example, an integrated serological profiling approach has been performed on purified IgG from participants of different HIV-1 vaccine trials.^2, 3^ By analyzing 6 Fc effector functions and 58 antigen-specific parameters, including IgG subtypes and Fcγ receptor interactions, it was found that the clear separation of the antibody signatures of different vaccine trials could be achieved and that unique humoral Fc-related signatures and differences that may be related to vaccine protection could be identified. These findings imply that the ‘systems serology' or ‘systems immunology' approaches have the potential to identify crucial immune signatures applicable to immune subtyping and subsequent precision medicine.

PIM will also benefit from a more detailed knowledge of the highly diverse human lymphocyte repertoire of B and T cells. Immune repertoires vary greatly not only among individuals but also in the same individual in response to changes in intrinsic (for example, aging) and environmental (for example, infection) factors.^4^ Due to the evolution of high-throughput sequencing techniques and related bioinformatic and statistical tools, some progress has recently been made in the deep profiling of repertoires of the B and T cell receptors, providing insight into the in-depth, sequence-based composition of immune repertoires.^5, 6, 7^ Understanding the composition and inter-individual diversity of immune repertoires would provide a framework upon which future studies can build to identify correlations between immune repertoire profiles and an individual's immune status, and as a result, translate such information into the delivery of precision medicine.

How will data from large precision medicine programs affect the future development of immunotherapies? Undoubtedly, knowledge of genomic data combined with phenotypes—currently being explored by companies such as Human Longevity—as well as a large quantity of additional information will significantly increase the efficacy and safety of immunotherapies. Currently, there are a number of such cases. For example, it is known that the antibody-dependent cellular cytotoxicity is affected by the CD16A allotype; this is why some therapeutic monoclonal antibodies (mAbs) have better performance when administered to CD16A 158V/V homozygous patients than to 158F/F patients.^8^ The frequency of the low-affinity allotype, 158F (0.6), is higher than that of the high-affinity allotype, 158V (0.4).^9^ Thus, to effectively recruit natural killer cells in all patient populations, it is important that anti-CD16A antibodies bind well to both allotypes. This consideration was taken into account when we identified new mAbs to CD16A, which bind equally both allotypes and made bispecific killer cell engagers targeting HIV-1.^10^ Another example comes from the hepatitis C virus (HCV), for which natural infection as well as the response to therapy were found to be associated with genetic variation in IL28B, explaining the considerable difference in response rates among different populations.^11^ These findings highlight the importance of immune responses in successful drug treatment and have the potential to improve treatment decisions for HCV-infected patients based on individual genotypes.

Individual responses in infectious diseases could vary even more than for other diseases because in many cases, the pathogen, for example, HIV-1, is itself highly variable, which allows the virus to evolve continuously and rapidly during the course of infection. The host immune response elicited by HIV-1 contributes to the repression of viral replication but also varies greatly in different individuals, and many recent studies have focused on the co-evolution of HIV-1 and humoral immune responses *in vivo*, which could guide the development of the HIV-1 vaccine and therapeutics.^12^ Therefore, in such cases, the availability of data for the specific pathogen, especially its sequence, combined with data from large databases would allow for the rapid prediction of possible responses to immunotherapies, especially by mAbs, which could be highly dependent on the type of pathogen isolated. In the future, the treatment of an infected person will involve the rapid identification of the pathogen combined with integration of the information for this individual's genetic, epigenetic and immunologic profiles to determine which immunotherapeutics are the most effective with lowest possible side effects. It would also be possible to tailor vaccines for certain groups of healthy individuals and significantly increase efficacy and markedly decrease side effects. Indeed, while marked differences exist in bulk IgG glycosylation among individuals that play a critical role in IgG Fc-mediated effector functions, it was found that different HIV-1 vaccines induced distinct vaccine-elicited antibody glycosylation profiles, indicating that antibody glycosylation can be tuned and programmed by certain vaccines in an antigen-specific manner.^13^

Another possible application of PIM to infectious diseases is the precision medicine approach to end the antimicrobial resistance crisis. Individual bacteria and viruses vary in their susceptibility to antimicrobial agents. The standard of care to treat some acute infections such as sepsis and ventilator-associated pneumonia may be based on empirical knowledge about the possible cause of infection, which contributes to the emergence and spread of antimicrobial resistance. In fact, even individuals of the same species may vary greatly in their susceptibility to antimicrobial agents.^14^ A potential PIM approach is based on the quick, reliable and relatively cheap diagnosis of the causative agent by the use of immunological reagents, for example, antibodies, followed by specific therapy for that agent based on mAbs or other immunology-related therapies if antibiotics are not available. This is especially important in light of the increasing occurrence of infections caused by multidrug-resistant microbes.

Similarly, the PIM approach for prevention and therapy of infections by emerging viruses would require the quick identification of the virus and its sequence if it is a new virus. This should be followed by using mAbs or other immunology-based agents specific for that virus. If the virus is newly identified, then new antibody-based therapeutics should be developed within weeks to months, which is now feasible due to advances in antibody-related technologies. The potential patients can be categorized based on their potential susceptibility to infection and disease. A good example is infections caused by the dengue virus, in which only ~0.5% of infected humans develop severe disease.^15^ Because typically immune-based therapies are currently expensive, such therapies could only be used in those patients who are predicted to develop disease.

The future of PIM and related efforts to develop biotherapies appears bright. It requires the combined efforts of governments and private companies as well as individual scientists to bring its concepts to fruition for the benefits of all.
